# Predicting saturated and unsaturated hydraulic conductivity of bentonite with data-driven and physics-guided machine learning models

**DOI:** 10.1038/s41598-026-66333-3

**Published:** 2026-08-18

**Authors:** Muntasir Shehab, Reza Taherdangkoo, Christoph Butscher

**Affiliations:** 1 Institute of Geotechnics, TU Bergakademie Freiberg, Gustav-Zeuner-Str. 1, Freiberg, 09599 Germany; 2 Freiberg Center for Water Research (ZeWaF), TU Bergakademie Freiberg, Akademiestr. 6, Freiberg, 09599 Germany

**Keywords:** Hydraulic conductivity, Bentonite, Boosting algorithm, Van Genuchten, Machine learning, Whale optimization algorithm, Engineering, Environmental sciences, Natural hazards, Solid Earth sciences

## Abstract

Accurate prediction of saturated and unsaturated hydraulic conductivity of bentonite is critical for assessing the long-term safety of high-level radioactive waste repositories, where barrier efficiency depends on coupled processes. This study develops a data-driven machine learning model to predict saturated hydraulic conductivity and a physics-guided machine learning model to predict unsaturated hydraulic conductivity of bentonite. For the saturated hydraulic conductivity prediction, a dataset of 215 experimental measurements was compiled, incorporating key soil properties such as montmorillonite content, specific gravity, liquid limit, plastic limit, initial water content, dry density, and temperature. To predict unsaturated hydraulic conductivity, the study integrates experimental data, synthetic data generated using the Van Genuchten model, and outputs from the developed machine learning model for saturated hydraulic conductivity. This dataset includes specific gravity, montmorillonite content, initial dry density, initial water content, initial void ratio, plasticity index, and suction. AdaBoost, CatBoost, and XGBoost algorithms were used to train the machine learning models, while the Whale Optimization Algorithm was applied for hyperparameter tuning. The developed models are publicly available via web-based interfaces for estimating saturated and unsaturated hydraulic conductivity of bentonite from soil parameters.

## Introduction

Hydraulic conductivity is a fundamental parameter governing water movement in soils. Bentonite is a particularly challenging material for determining hydraulic conductivity due to its distinctive physical characteristics, such as the presence of montmorillonite and extremely fine particles. Water transport in bentonite occurs through a dual-pore system comprising micropores and nanopores. In these pores, adsorption and surface forces govern fluid flow, which can lead to deviations from Darcy’s law^1,2^. Furthermore, the presence of montmorillonite leads to pronounced swelling due to interlayer hydration.

Swelling pressure alters the pore structure and further complicates flow pathways. The resulting hydraulic behaviour, governed by coupled hydraulic and mechanical processes, is nonlinear and hysteretic, making it difficult to predict using conventional models^3^. These complexities are particularly critical when bentonite is used as a sealing or buffer material in high-level radioactive waste repositories, where its hydraulic behaviour must be evaluated under variable suction conditions.

Saturated hydraulic conductivity is typically measured using constant-head, falling-head, or air-entry permeameter permeability tests, which provide reliable and direct values^4^. In cases where experimental determination is not feasible, predictive approaches such as the Kozeny–Carman equation are often used^5^. This equation yields reasonable estimates for sandy and granular soils, where its assumptions about pore geometry and flow are valid. However, its application to bentonite is limited, as it neglects nanoscale pores, surface interactions, and physicochemical effects that strongly govern water flow in fine-grained materials.

Unsaturated hydraulic conductivity can be determined, e.g., via the instantaneous-profile or evaporation methods, but such tests are challenging due to the difficulty of controlling and maintaining suction in the laboratory^6^. Consequently, a common practice is to combine an experimentally measured or predicted saturated hydraulic conductivity with an empirical model to estimate the unsaturated hydraulic conductivity. Among these models, the Van Genuchten–Mualem formulation^7^is widely adopted (see also, e.g^8^.,). In this approach, the saturated hydraulic conductivity is coupled with the soil water retention curve to compute effective saturation and a relative permeability function, from which the unsaturated hydraulic conductivity can be obtained^9^.

These empirical models are developed by fitting predefined functional forms to experimental data, which makes them practical for different soil types. However, the application of empirical models to bentonite is difficult, particularly for predicting unsaturated hydraulic conductivity. These formulations typically assume a continuous pore-size distribution and overlook the tight hydro-mechanical coupling that controls pore structure evolution in swelling clays. Furthermore, these models demand extensive calibration, and their predictive performance typically deteriorates when applied outside the calibration domain^10,11^.

In recent years, data-driven machine learning approaches have emerged as promising alternatives to direct measurement and empirical estimation of bentonite properties. For instance^3^, developed a data-driven machine learning model to predict the maximum swelling pressure of bentonite and bentonite mixtures. Similarly^12^, developed machine learning models to predict the hydraulic conductivity of unsaturated compacted bentonite. As long as sufficient experimental coverage is available, fully data-driven models remain accurate, such as for saturated hydraulic conductivity.

However, using sparse measurement data may produce physically inconsistent predictions. This limitation becomes more pronounced in applications where a variable must be evaluated across a wide range of conditions/scenarios. For instance, determining the hydraulic response of soil from an initial, i.e. low-suction, condition to high suction requires measurements at multiple suction intervals, which are labor-intensive, costly, and difficult to maintain under consistent laboratory conditions. To address such challenges^13^, developed a physics-based machine learning model to predict the soil water retention curve (SWRC) of compacted bentonite. By combining 46 bentonite soil datasets with augmented data generated from fitted Van Genuchten models, the study showed that machine learning model can reliably capture the water retention behaviour across a wide range of soil conditions. Despite the advantages of machine learning and the successful use of augmented data to represent complex hydraulic responses of bentonite across wide suction ranges, a dedicated physics-guided machine learning strategy that explicitly links saturated and unsaturated hydraulic conductivity remains absent.

This study addresses that gap by developing a machine learning model that uses a physics-guided data augmentation technique to predict both the saturated and unsaturated hydraulic conductivity of bentonite within a unified framework. First, a data-driven machine learning model is trained to predict saturated hydraulic conductivity from key soil index properties. The predicted saturated conductivity is then embedded into the Van Genuchten formulation to describe unsaturated hydraulic behaviour through its linkage with the soil water retention relationship. Synthetic hydraulic conductivity data are generated from the fitted Van Genuchten model to enforce physical continuity across suction states. These physics-guided synthetic data are combined with soil index parameters and used to train the final machine learning model, enabling the model to learn both empirical soil-property relationships and the governing physical constraints simultaneously.

The machine learning models were trained using AdaBoost, CatBoost, and XGBoost algorithms, while hyperparameter tuning was performed using the Whale Optimization Algorithm. This novel methodology enhances the reliability of hydraulic conductivity predictions for bentonite and contributes to improved safety assessments of engineered barrier systems for high-level radioactive waste disposal. In addition, practical application of such predictive models often remains limited by the need for specialized software tools and programming expertise. To address this limitation, the developed models in this study are deployed through user-friendly web-based interfaces that enable direct estimation of hydraulic conductivity from soil parameters. This deployment allows practitioners to obtain reliable predictions without requiring laboratory testing or prior experience in coding or machine learning.

## Data

This study used two independent datasets to develop machine learning models to predict saturated and unsaturated hydraulic conductivity. For simplicity, the term “saturated hydraulic conductivity” is designated as $K_s$, and “unsaturated hydraulic conductivity” is designated as *K(θ )*, while the full terminology is used where appropriate. Subchapter 2.1 presents a statistical discussion of the compiled $K_s$ dataset, while a brief review of the associated experimental studies is provided in the Supplementary Material. The dataset of the *K(θ )* is a combination of experimental data of soil water retention curve (SWRC), predicted $K_s$ data, and synthetic data from Van Genuchten model (described later in Chapter 3.1). As a short review of the experimental studies of SWRC with a statistical discussion is already available at^13^, only a brief review of *K(θ )* dataset is provided in Subchapter 2.2.

### Saturated hydraulic conductivity

A dataset of 215 data points was compiled from published experimental studies on the $K_s$ of bentonite^14–25^. The statistical characteristics of the dataset, summarized in Table 1, present the distributions of key soil parameters used in this study. The dataset comprises bentonite samples, each described by montmorillonite content ($\text {Mt.c}$,%), liquid limit ($w_\text {L}$,%), plastic limit ($w_\text {p}$,%), specific gravity ($G_\text {s}$,-), initial water content ($w_\text {i}$,%), temperature ($T,^\circ \hbox {C}$), dry density ($\rho _\text {d}$, $\hbox {g cm}^{-3}$), and saturated hydraulic conductivity ($K_s$, $\hbox {m s}^{-1}$).

**Table 1:** Statistical analysis of 215 data collected for developing machine learning model to predict $K_s$.

	$\text {Mt.c}$ (%)	$w_\text {L}$(%)	$w_\text {p}$(%)	$G_\text {s}$ (-)	$w_\text {i}$ (%)	$\hbox {T} (^\circ \hbox {C}$)	$\rho _\text {d}$ ($\hbox {g cm}^{-3}$)	$K_s$ ($\hbox {m s}^{-1}$)
mean	60	255	49	2.67	14	24	1.46	$3.76 \times 10^{-10}$
std	24	160	7.37	0.1	8	13	0.31	$1.34 \times 10^{-9}$
min	9	99	27	2.4	6	20	0.6	$4.2 \times 10^{-15}$
max	95	616	60	2.78	36	150	2.00	$1.06 \times 10^{-8}$
skew	*-0.19*	0.86	*-0.67*	*-1.43*	2.02	6.75	*-0.53*	0.37
kurtosis	*-0.82*	*-0.61*	0.53	1.11	3.15	54.18	*-0.15*	*-1.14*

The montmorillonite content ($\text {Mt.c}$) shows a mean of *60%* with a standard deviation of *24%* and a minimum value of *9%*. This wide range indicates that some samples contained bentonite only as a secondary component, showing that the dataset includes mixtures prepared with varying bentonite proportions. The liquid limit ($w_\text {L}$) and plastic limit ($w_\text {P}$) values confirm the high plasticity of most samples, which is well known characteristic of bentonite. The mean specific gravity ($G_s = 2.67$) with a narrow standard deviation (0.1) aligns well with typical mineralogical values known for bentonite.

A mean value of *14%*, a standard deviation of *8%*, and a positive skew value of 2.02, the initial water content ($w_\text {i}$) indicates that most samples were tested in relatively dry conditions; however, hydration effects were also examined in a few cases. The temperature (*T*) ranges from $20^\circ \hbox {C}$ to $150^\circ \hbox {C}$, but mean value of $24^\circ \hbox {C}$ with a high kurtosis value (54.18), showing that while most experiments were conducted under laboratory conditions, few samples were tested under varying temperature conditions to explore the thermal effects on hydraulic behaviour. Dry density ($\rho _\text {d}$) values vary between 0.6 and 2.0 g/cm^3^, with a mean of 1.46 g/cm^3^ and standard deviation of 0.31. This range reflects different compaction levels, from loosely packed to densely compacted specimens. Such variability captures the influence of compaction energy on the physical structure of the bentonite matrix.

Saturated hydraulic conductivity ($K_s$) spans approximately seven orders of magnitude, from $4.2 \times 10^{-15}$ $\hbox {m s}^{-1}$ to $1.06 \times 10^{-8}$ $\hbox {m s}^{-1}$. This large variation reflects the strong dependency of $K_s$ on mineral composition and dry density. Lower $K_s$ values are typically associated with higher montmorillonite content and greater compaction, implying reduced pore connectivity and limited fluid transmission. Conversely, higher $K_s$ values correspond to lower density or bentonite mixtures, where larger pore spaces facilitate higher permeability. Such a broad spread in $K_s$ values indicates that the dataset captures a wide range of hydraulic behaviours, making it well-suited for developing a machine learning model capable of achieving reliable and robust predictive performance.

### Unsaturated hydraulic conductivity

^12^ highlighted that direct measurements of unsaturated hydraulic conductivity of bentonite are scarce. To ensure a dataset that covers a broad range of *K(θ )* values over a wide range of suction, instead of relying solely on scarce direct measurements, a physic guided modeling approach was adopted. In this process, the *K(θ )* dataset was obtained using the Van Genuchten model, where experimental SWRC data was required. This study used 46 SWRC data, including 311 direct measurements of water content at different suctions, compiled earlier by^13^.

A detailed description of the experimental methodologies, suction control techniques, temperature conditions, and statistical characteristics of the dataset is provided in^13^. Table 2 provides a concise overview of the dataset composition used in the present study in terms of bentonite type, boundary condition, and hydraulic path. The compiled dataset includes SWRCs obtained under both confined and unconfined boundary conditions and along wetting and drying paths, thereby capturing swelling-controlled water retention behaviour and hydraulic hysteresis characteristic of bentonite. Each SWRC is associated with a consistent set of soil physical and mineralogical parameters, including specific gravity, dry density, initial water content, initial void ratio, plasticity index, and montmorillonite content. Binary indicators were introduced in dataset to distinguish confined versus unconfined conditions and wetting versus drying paths.

To ensure internal consistency of the compiled dataset, missing soil parameters reported in the original experimental studies were reconstructed where feasible using standard soil mechanics relationships. For example^32^, did not report the initial void ratio ($e_i$), which was therefore calculated from the specific gravity, water density, and dry density following classical formulations^37^. Similarly, the plasticity index (($I_\text {p}$) was derived from the reported liquid and plastic limits when not explicitly provided. For bentonite mixtures (e.g^35^.,), the montmorillonite content was determined by multiplying the bentonite proportion in the mixture by the montmorillonite percentage of the original bentonite material.

**Table 2:** Overview of the compiled SWRC dataset used in this study, showing the number of datasets by bentonite type, boundary condition, hydraulic path, and corresponding references. Detailed experimental protocols and individual test conditions are reported in^13^.

Bentonite type	SWRCs	Confined		Unconfined		Reference(s)
		Wetting	Drying	Wetting	Drying	
FEBEX bentonite	18	10	4	3	1	^26,27^
MX-80	11	7	4	0	0	^28–30^
GMZ/GMZ01	4	2	1	1	0	^31–33^
Kunigel bentonite–sand mixture	3	1	1	1	0	^34^
Calcigel bentonite–sand mixture	5	5	0	0	0	^35^
Bentonite–silt mixture	5	1	4	0	0	^36^
Total	46	26	14	6	1	

## Methodology

### Workflow

**Fig. 1:**
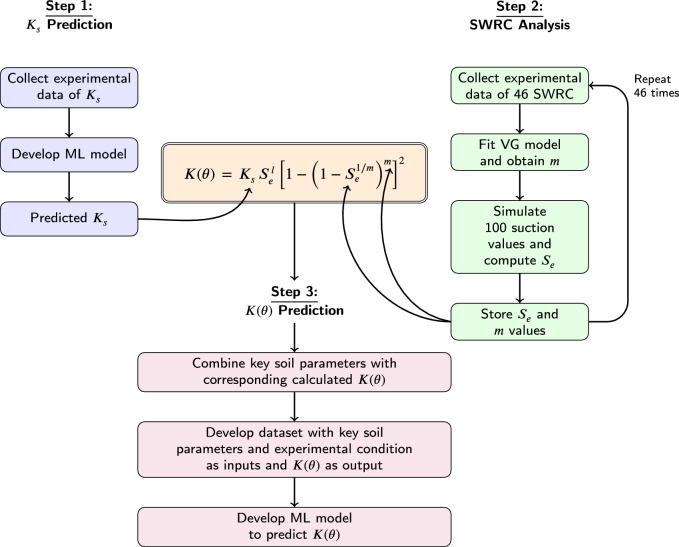
Workflow of this study for *K(θ )* prediction using experimental data, SWRC analysis, and ML-based modeling.

The workflow has three sequential steps (Figure 1). To predict the saturated hydraulic conductivity ($K_s$), a data-driven approach was adopted by obtaining data from experimental measurements. In contrast, the prediction of the unsaturated hydraulic conductivity, *K(θ )*, was based on a combination of experimental data, predicted value of $K_s$ from the developed machine learning model, and synthetic data generated using the Van Genuchten model. The Van Genuchten model is defined as^7^:

1 $$\begin{aligned} K(\theta ) = K_{s} \, S_{e}^{l} \, \left[ 1 - \left( 1 - S_{e}^{1/m} \right) ^{m} \right] ^{2} \end{aligned}$$

where *K(θ )* represents the unsaturated hydraulic conductivity, $K_{s}$ denotes the saturated hydraulic conductivity, $S_{e}$ signifies the effective saturation, *m* is a fitting parameter, and *l* represents the pore-connectivity parameter (typically set to *l = 0.5*). The effective saturation $S_{e}$ is defined as^7^:

2 $$\begin{aligned} S_{e} = \frac{\theta - \theta _{r}}{\theta _{s} - \theta _{r}} \end{aligned}$$

Figure 1, shows the three steps of the workflow detailed below.

Step 1. Prediction of saturated hydraulic conductivity ($K_s$): A dataset of 215 experimental measurements was compiled from existing literature, incorporating soil index properties and corresponding $K_s$ values. This dataset was used to develop a machine learning models for predicting $K_s$.
Step 2. Determination of soil water retention parameters: A separate dataset comprising 311 experimental measurements corresponding to 46 soil water retention curves (SWRCs) for bentonite was utilized from^13^. The van Genuchten model for SWRC expressed as:
  3 $$\begin{aligned} \theta (\psi ) = \theta _r + \frac{\theta _s - \theta _r}{\left( 1 + \left( \alpha |\psi |\right) ^n\right) ^m}, \end{aligned}$$
  where $\theta _s$ and $\theta _r$ are the saturated and residual water contents, respectively, and $m = 1 - \frac{1}{n}$. The optimal value of the parameters ($\theta _s$, $\theta _r$, *α*, *n*, and *m*) obtained during fitting process. For the optimization task, the Levenberg–Marquardt^38,39^ algorithm was employed following^13^, with the exception that an additional constraint was imposed requiring *n* > 1.1. The Van Genuchten model was fitted to these 46 SWRCs, resulting 46 sets of optimal values of the model parameters. The optimized parameters ($\theta _s$ and $\theta _r$) obtained from each curve were used to calculate the effective saturation ($S_e$).
Step 3. Prediction of unsaturated hydraulic conductivity (*K(θ )*): Using the predicted $K_s$ values from Step 1 and the calculated $S_e$ and *m* parameters from Step 2, Equation 1 was employed to compute *K(θ )* for 46 soil samples. For each soil water retention curve, *K(θ )* was calculated at 100 randomly generated suction values, resulting in a total of 4,600 *K(θ )* predictions. The randomly generated suction values ensure adequate coverage of the low-suction (near-saturated), transition, and high-suction regions. The calculated *K(θ )* values were then combined with the corresponding experimental conditions and soil parameters to form a dataset for developing the ML model for *K(θ )* prediction.

Although the *K(θ )* values were generated using the Van Genuchten model, they were derived from the diverse set of experimentally measured SWRCs presented in this study. The experimental procedures, statistical analyses, training algorithms, hyper-parameter optimization, and performance evaluation of the machine learning models are discussed in subsequent chapters. The complete set of optimized Van Genuchten parameters is provided in the supplementary materials.

### Training algorithms

In this study, three gradient boosting algorithms were employed to predict the hydraulic conductivity of bentonite. These algorithms are based on the principle of ensemble learning, whereby weak learners are sequentially combined to minimize prediction error through iterative optimization.

#### Adaptive boosting

Adaptive Boosting (AdaBoost) is an ensemble learning technique that constructs a strong predictive model by sequentially combining multiple weak regression learners^40^. The algorithm assigns adaptive weights to training samples, allowing subsequent learners to focus progressively on observations associated with larger prediction errors. This iterative reweighting strategy enhances the model’s ability to capture complex nonlinear relationships. The final ensemble prediction is obtained as a weighted sum of the individual weak learners:

4 $$\begin{aligned} F(x) = \sum _{m=1}^{M} \alpha _m \, h_m(x) \end{aligned}$$

where *M* denotes the total number of weak learners, $h_m(x)$ represents the prediction of the $m^{\text {th}}$ learner, and $\alpha _m$ is the corresponding contribution weight. At each boosting iteration, the performance of the weak learner is evaluated using a weighted regression loss function *L(· )*, leading to the definition of the normalized prediction error:

5 $$\begin{aligned} \varepsilon _m = \frac{\sum _{i=1}^{N} W_i \, L\!\left( y_i, h_m(x_i)\right) }{\sum _{i=1}^{N} W_i} \end{aligned}$$

where $w_\text {i}$ is the weight of the $i^{\text {th}}$ sample and $y_i$ denotes the observed target value. Based on this error measure, the learner weight is computed as:

6 $$\begin{aligned} \alpha _m = \frac{1}{2} \ln \left( \frac{1 - \varepsilon _m}{\varepsilon _m} \right) \end{aligned}$$

The sample weights are then updated to emphasize data points with larger prediction errors:

7 $$\begin{aligned} W_i \leftarrow W_i \, \exp \left( \alpha _m \, L\!\left( y_i, h_m(x_i)\right) \right) \end{aligned}$$

followed by normalization to ensure $\sum _i W_i = 1$. Through this adaptive weighting mechanism, AdaBoost incrementally minimizes the overall regression loss, leading to improved predictive accuracy and robustness across heterogeneous datasets.

#### Extreme gradient boosting

Extreme Gradient Boosting (XGBoost) represents an advanced implementation of the gradient boosting regression tree (GBRT) framework^41,42^. By reducing the complexity of individual regression trees and introducing an efficient tree-boosting framework, XGBoost aligns closely with the principles of regularized learning. Two primary mathematical extensions distinguish XGBoost from conventional GBRT, establishing it as a highly effective ensemble technique for consolidating multiple weak learners into a robust predictive model.

First, XGBoost incorporates an objective function consisting of a loss function and a regularization term into the standard GBRT architecture. This objective function is expressed as:

8 $$\begin{aligned} Obj = \sum _{i} L(y_i, {\hat{y}}_i) + \sum _{k} \Omega (f_k) \end{aligned}$$

where $L(y_i, {\hat{y}}_i)$ is a convex differentiable loss function that quantifies the difference between the predicted and actual values of the training data. The term $\Omega (f_k)$ describes the complexity of the *k*-th tree and is defined as:

9 $$\begin{aligned} \Omega (f_k) = \gamma T + \frac{1}{2} \lambda \Vert w\Vert ^2 \end{aligned}$$

In this formulation, *T* denotes the number of leaves and *w* represents the leaf weights. To reduce overfitting, the inclusion of $\Omega (f_k)$ incentivizes the optimization of simpler trees while simultaneously minimizing the loss function $L(y_i, {\hat{y}}_i)$. The parameters *γ* and *λ* are adjustable constants; *λ* penalizes extreme weights via *L*2 regularization, while *γ* provides a constant penalty for each leaf added to the tree. Furthermore, to support a broader range of loss functions, the algorithm utilizes a second-order Taylor expansion for optimization, whereas standard GBRT relies only on the first-order expansion^41,43^.

#### Categorical boosting

The categorical boosting algorithm (CatBoost) is a gradient boosting framework specifically designed to efficiently handle categorical and tabular datasets^44^. CatBoost introduces the concept of ordered boosting, which reduces target leakage and prediction bias during training. It is particularly suitable for datasets where categorical differentiation plays a significant role.

To manage categorical features, CatBoost employs target-based statistics that convert categorical variables into numerical representations while preserving their statistical relationships with the target variable. The overall prediction of CatBoost is expressed as:

10 $$\begin{aligned} {\hat{y}} = \sum _{k=1}^{K} \lambda _k \, T_k(x) \end{aligned}$$

where ${\hat{y}}$ denotes the predicted output, $T_k(x)$ represents the $k^{\text {th}}$ decision tree, $\lambda _k$ is the learning rate that regulates the contribution of each tree, and *K* is the total number of trees in the ensemble. By iteratively fitting new trees to the residuals of prior models, CatBoost achieves strong generalization performance while effectively reducing overfitting.

### Optimization algorithm

The whale optimization algorithm (WOA), is a nature-inspired metaheuristic based on the bubble-net hunting strategy of humpback whales^45^. WOA alternates between two main phases: exploitation, represented by encircling and spiral movements around the best-known solution, and exploration, represented by random searches across the solution space.

Let ${\textbf{X}}_i(t)$ denote the position of the *i*th whale at iteration *t*, and ${\textbf{X}}^*(t)$ the best current solution. The encircling mechanism is defined as:

11 $$\begin{aligned} {\textbf{D}} = |{\textbf{C}} \cdot {\textbf{X}}^*(t) - {\textbf{X}}_i(t)|, \quad {\textbf{X}}_i(t+1) = {\textbf{X}}^*(t) - {\textbf{A}} \cdot {\textbf{D}}, \end{aligned}$$

where ${\textbf{A}} = 2a \cdot {\textbf{r}}_1 - a$ and ${\textbf{C}} = 2 \cdot {\textbf{r}}_2$. Here, ${\textbf{r}}_1$ and ${\textbf{r}}_2$ are random vectors in [0, 1], and *a* decreases linearly from 2 to 0 as iterations progress.

The spiral updating behaviour, which simulates the bubble-net motion, is given by:

12 $$\begin{aligned} {\textbf{X}}_i(t+1) = |{\textbf{X}}^*(t) - {\textbf{X}}_i(t)| e^{b l} \cos (2\pi l) + {\textbf{X}}^*(t), \end{aligned}$$

where *b* defines the spiral shape and *l ∈ [-1,1]* is a random number.

Exploration occurs when $|{\textbf{A}}| \ge 1$, allowing the whale to move relative to a randomly selected solution ${\textbf{X}}_{\text {rand}}(t)$:

13 $$\begin{aligned} {\textbf{X}}_i(t+1) = {\textbf{X}}_{\text {rand}}(t) - {\textbf{A}} \cdot |{\textbf{C}} \cdot {\textbf{X}}_{\text {rand}}(t) - {\textbf{X}}_i(t)|. \end{aligned}$$

A random probability *p ∈ [0,1]* determines whether the whale performs encircling (*p < 0.5*) or spiral motion (*p \ge 0.5*). The algorithm iteratively updates whale positions until the stopping condition is met, and the best solution ${\textbf{X}}^*(t)$ is considered optimal. Owing to its simple structure, global search capability, and low parameter dependency, WOA has been successfully applied to various engineering optimization and machine learning problems^46,47^.

### Machine learning model training

A structured and carefully designed training strategy was adopted to develop reliable machine learning models to predict *K(θ )* and $K_s$. In total nine input parameters were used to predict *K(θ )*. The confined/unconfined conditions and wetting/drying paths were encoded as binary labels, while the remaining inputs corresponded to fundamental soil properties, including specific gravity ($G_\text {s}$), dry density ($\rho _\text {d}$), montmorillonite content ($\text {Mt.c}$), initial water content ($w_\text {i}$), initial void ratio ($e_i$), plasticity index (($I_\text {p}$), and suction (*ψ*). To predict $K_s$, seven input parameters were used, including montmorillonite content ($\text {Mt.c}$), liquid limit ($w_\text {L}$), plastic limit ($w_\text {P}$), specific gravity ($G_\text {s}$), initial water content ($w_\text {i}$), temperature (*T*), and dry density ($\rho _\text {d}$). A common strategy in machine learning is to randomly divide the dataset into training and testing subsets to reduce overfitting and evaluate generalization performance^48^. To predict $K_s$, 215 data were randomly divided using a 80:20 ratio, meaning that 43 samples were allocated randomly as unseen data to evaluate the performance of trained model.

It was initially assumed that this strategy might not be suitable for predicting *K(θ )*, since the dataset contains 46 SWRC measurements, and each SWRC was used to generate a *K(θ )* curve at different suction levels. Each curve has its own associated soil parameters, which remain constant along the curve, with only suction and the corresponding *K(θ )* values varying. Applying the same 80:20 separation strategy was therefore considered inappropriate for the initial *K(θ )* prediction approach. Moreover, allocating 37 subsets for training and 9 for testing introduced a potential imbalance because only 7 experiments were conducted under unconfined conditions. A fully random split could thus bias the training set toward confined conditions, limiting the model’s ability to generalize across both scenarios.

To overcome this issue, a binary labeling strategy was implemented for the augmented dataset. Of the 4, 600 total data points, 3, 900 corresponded to confined tests and were labeled as 1, while 700 data points represented unconfined tests and were labeled as 0. Likewise, the hysteresis information was captured through the drying and wetting paths: 3, 000 data points corresponded to the wetting path (label 1), and 1, 600 corresponded to the drying path (label 0). After labeling, the dataset was suitable for applying the same 80:20 random separation strategy for training and testing subsets. With both datasets prepared, the three boosting algorithms mentioned above were trained in a consistent manner. The target variables of both datasets, *K(θ )* and $K_s$, were placed in the final column of separated CSV files to ensure a standardized and automated workflow. Prior to model training, all conductivity values were transformed using a base–10 logarithm, i.e., $K \rightarrow \log _{10}(K)$.

A critical part of model training was hyperparameter optimization. For each of the three training algorithms (AdaBoost, XGBoost and CatBoost), a set of hyperparameters was selected initially through trial-and-error. Afterward, the lowest feasible value of each hyperparameter was assigned as the lower bound, and the highest feasible value was assigned as the upper bound. To determine the optimal configuration of hyperparameter value within these assigned bounds, the metaheuristic algorithm WOA was employed. The WOA was configured with a population size of 20 and 20 iterations following initial trial-and-error experiments. While optimizing hyperparameters, a 5-fold cross-validation scheme was applied to ensure reliable evaluations. In this procedure, the training dataset is divided into five subsets. In order to evaluate each candidate hyperparameter set explored by the WOA, the model is trained on four subsets and validated on the remaining subset, which provides an unbiased and stable estimate of model performance. The final optimized models were evaluated using 20 percent of the data as a test set and the best performing model among the three selected algorithms was considered the optimal model for each prediction task.

## Results

The optimization process obtained 300 sets of optimized hyperparameter values correspond to the *K(θ )* dataset (i.e., 100 per algorithm). Among the 300 models, the best-performing model was selected based on the RMSE and $R^2$ values of the test set. The hyperparameter spaces, their optimal values, and the corresponding best model performances of the three boosting algorithms, evaluated in terms of RMSE, MSE, and $R^2$ for predicting both $K_s$ and *K(θ )*, are summarized in Table 3. As the target values were transformed to $\log _{10}(K)$ prior to training, the reported MSE and RMSE quantify errors in logarithmic space, i.e., in terms of orders of magnitude rather than physical conductivity units.

Following the same selection procedure, the best model was obtained for the $K_s$ dataset. At the end, only the two best-performing models from each prediction task were retained for subsequent analysis. For $K_s$, all three algorithms (AdaBoost, XGBoost, and CatBoost) achieve high predictive accuracy with very small discrepancies between training and testing performance. This indicates robust generalization and confirms that saturated hydraulic conductivity can be modeled reliably within the adopted framework.

A similar trend is observed for the *K(θ )* dataset. None of the algorithms exhibits severe overfitting, as evidenced by small differences between training and testing RMSE values and comparable R^2^ scores for both subsets. Despite the structured nature of the unsaturated dataset, which consists of 4, 600 rows generated by pairing soil parameters with 46 SWRC, all optimized models are able to generalize beyond the training data. This shows that the unified optimization strategy is suitable for developing machine learning model for saturated and unsaturated conductivity prediction.

Nevertheless, clear differences appear in terms of predictive consistency and overall accuracy. For both $K_s$ and *K(θ )*, CatBoost shows best performance as it yields the lowest test-set RMSE and maintains the most balanced performance between training and testing. XGBoost performs competitively but shows slightly larger generalization gaps and higher test errors, particularly for *K(θ )*. AdaBoost, while still providing reasonable predictions, is comparatively weaker for the unsaturated case, showing larger errors than the other two algorithms, although its train–test gap remains small.

The superior stability of CatBoost can be attributed to its ordered boosting strategy and its intrinsic regularization mechanisms, such as random strength and bagging temperature. These features reduce the tendency to fit spurious patterns in structured or partially imbalanced datasets and promote consistent generalization. While the other boosting methods could be further tuned to achieve similar behaviour, the objective of this study is not to equalize algorithmic performance through extensive manual intervention, but rather to identify the model that naturally delivers robust and consistent predictions across distinct dataset types under a unified training and optimization framework.

Based on these considerations, CatBoost emerges as the most reliable model for predicting both saturated and unsaturated hydraulic conductivity. Its consistently low test errors and stable train–test behaviour across $K_s$ and *K(θ )* justify its selection as the preferred model for subsequent analyses and interpretation. From a physical perspective, the generally higher prediction accuracy observed for saturated hydraulic conductivity reflects the more deterministic control of pore structure and connectivity under fully saturated conditions. In contrast, *K(θ )* is inherently more variable because it is strongly influenced by suction and degree of saturation, introducing additional nonlinearity and heterogeneity. The ability of CatBoost to maintain stable generalization under these contrasting regimes further reinforces its suitability for modeling nonlinear and multivariate soil hydraulic behaviour.

In summary, although all investigated boosting algorithms perform well, CatBoost provides the most robust and generalizable predictions across both saturated and unsaturated conditions. This makes it the preferred model for the continuation of the study, particularly for integration into numerical simulations and coupled hydro-mechanical modeling frameworks.

**Table 3:** Boundaries, optimal hyperparameters, and model performance for boosting algorithms in predicting saturated ($K_s$) and unsaturated (*K(θ )*) hydraulic conductivity of bentonite. Metrics are based on the specific model runs provided for each phase.

**Algorithm**	**Hyperparameter**	**Lower**	**Upper**	**Optimal Value**		**Target**	**Phase**	**Performance Metrics**		
				$K_s$	*K(θ )*			$R^2$	$\hbox {MSE}^{b}$	$\hbox {RMSE}^{b}$
CatBoost	Iterations	1	1000	497.9433	677.7912	$K_s$	Train	0.9959	0.1155	0.3399
	Learning rate (*η*)	0.0001	1.0	0.1392	0.0740		Test	0.9922	0.2534	0.5033
	Depth	1	15	4.0018	6.1999	*K(θ )*	Train	0.9954	0.0337	0.1837
	Random strength	0.0	10.0	6.4675	5.5428		Test	0.9940	0.0409	0.2022
	Bagging temperature	0.0	10.0	3.3603	$0.0^{c}$					
XGBoost	Number of estimators	1	1000	853.6258	419.1278	$K_s$	Train	0.9527	1.4525	1.2052
	Learning rate (*η*)	0.0001	1.0	0.1913	0.6232		Test	0.9526	1.0849	1.0416
	Max Depth	1	20	5.2277	19.2338	*K(θ )*	Train	0.9903	0.0714	0.2671
	Subsample ratio	0.1	1.0	0.3825	0.3972		Test	0.9827	0.1230	0.3508
	Column sample by tree	0.1	1.0	0.9011	0.8149					
	Min child weight	0	15	4.3987	13.1521					
	Gamma (*γ*)	0	10	2.8307	0.1342					
	Reg_lambda (*λ*)	0	10	5.4364	4.7834					
	Reg_alpha (*α*)	0	10	7.0572	2.2203					
AdaBoost	Number of estimators	1	1000	510.1858	52.7894	$K_s$	Train	0.9418	1.7590	1.3263
	Learning rate (*η*)	0.0001	2.0	0.0475	1.1486		Test	0.9327	1.6717	1.2929
	Loss$\hbox {function}^{a}$	0	2	1	2	*K(θ )*	Train	0.9728	0.2094	0.4576
	Max Depth (Base)	1	10	–	1.9995		Test	0.9630	0.2128	0.4614

^a^Loss function mapping: 0 = linear, 1 = square, 2 = exponential. ^b^MSE, RMSE are computed in$\log _{10}(K)$space. The corresponding average error factor in physical units is given by$10^{\text {RMSE}}$. ^c^ For*K(θ )*, the optimization algorithm selected no bagging temperature, meaning the best performing model was achieved without any stochastic resampling.

### Comparative analysis of machine learning model in predicting $K_s$

**Fig. 2:**
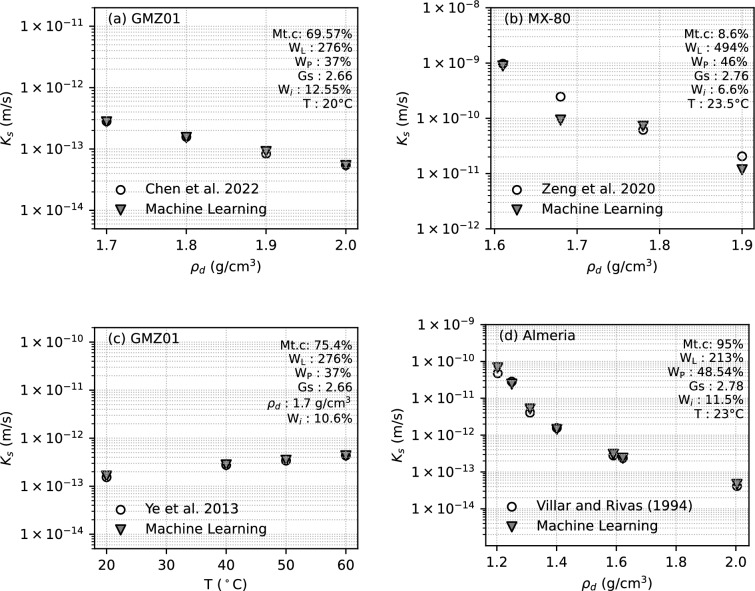
Performance analysis of machine learning model in predicting $K_{s}$: (**a**) GMZ01 bentonite^15^; (**b**) MX-80 bentonite^25^; (**c**) GMZ01 bentonite^24^; (**d**) Almeria bentonite^22^.

^22^ determined the $K_s$ of Almería bentonite, which has a high montmorillonite content of approximately 95 percent. The developed machine learning model shows high accuracy in predicting $K_s$. However, accuracy decreases slightly at lower dry densities. The mean value of dry density during the training of the model was 1.46, as shown in Table 1. At low compaction, the microstructure and pore connectivity of bentonite are altered, and the model also captures the behaviour, though with slightly lower accuracy. Overall, the developed machine learning model shows consistency in predicting $K_s$ values of bentonite across a wide range of key soil parameters and conditions. The performance of the model drops noticeably only when the montmorillonite content becomes very low and approaches the limits of the training domain.

The performance of the developed machine learning model was evaluated by comparing experimental and predicted values of $K_s$ (Figure 2). Figure 2a shows that the predictions are consistent with the experimental values of^15^ of GMZ01 bentonite at different dry densities. However, as shown in Figure 2b the machine learning model shows deviation in predicting the $K_s$. Zeng et al.^25^ conducted experiments at different dry densities of the samples where the samples contained comparatively lower montmorillonite content (only *8.6%*). Since montmorillonite strongly governs the microstructure and pore connectivity of compacted bentonites, a reduced clay fraction results in lower swelling capacity and higher variability of $K_s$.

The model was trained on data with a mean montmorillonite content of 60%. The value of 8.6% therefore lies at the lower bound of the training data range summarized in Table 1. The deviation is visibly high when the dry density is close to 1.7 g/cm^3^. However, Figure 2c shows that the model is performing well in predicting $K_s$ at the dry density of 1.7 g/cm^3^. The observed predictive deviations for MX-80 bentonite (Figure 2b) primarily arise from its low montmorillonite content, which places it at the edge of the training domain. These deviations do not reflect any structural limitation of the model itself.

Figure 2c also shows that the developed machine learning model is capable of capturing the temperature dependence of $K_s$ values. An increase in temperature reduces the viscosity of the pore fluid and alters the clay microstructure, which collectively lead to an enhancement in hydraulic conductivity. Temperature is sparsely represented in the collected data. Nevertheless, the model performs well because the physical influence of temperature on $K_s$ is predominantly monotonic and less complex than the mineralogical controls associated with the montmorillonite content. In contrast, variations in montmorillonite content induce nonlinear microstructural changes. These changes occupy a far broader and more heterogeneous region of the feature space, making sparse data in that domain more consequential.

### Comparative analysis of machine learning model in predicting *K(θ )*

**Fig. 3:**
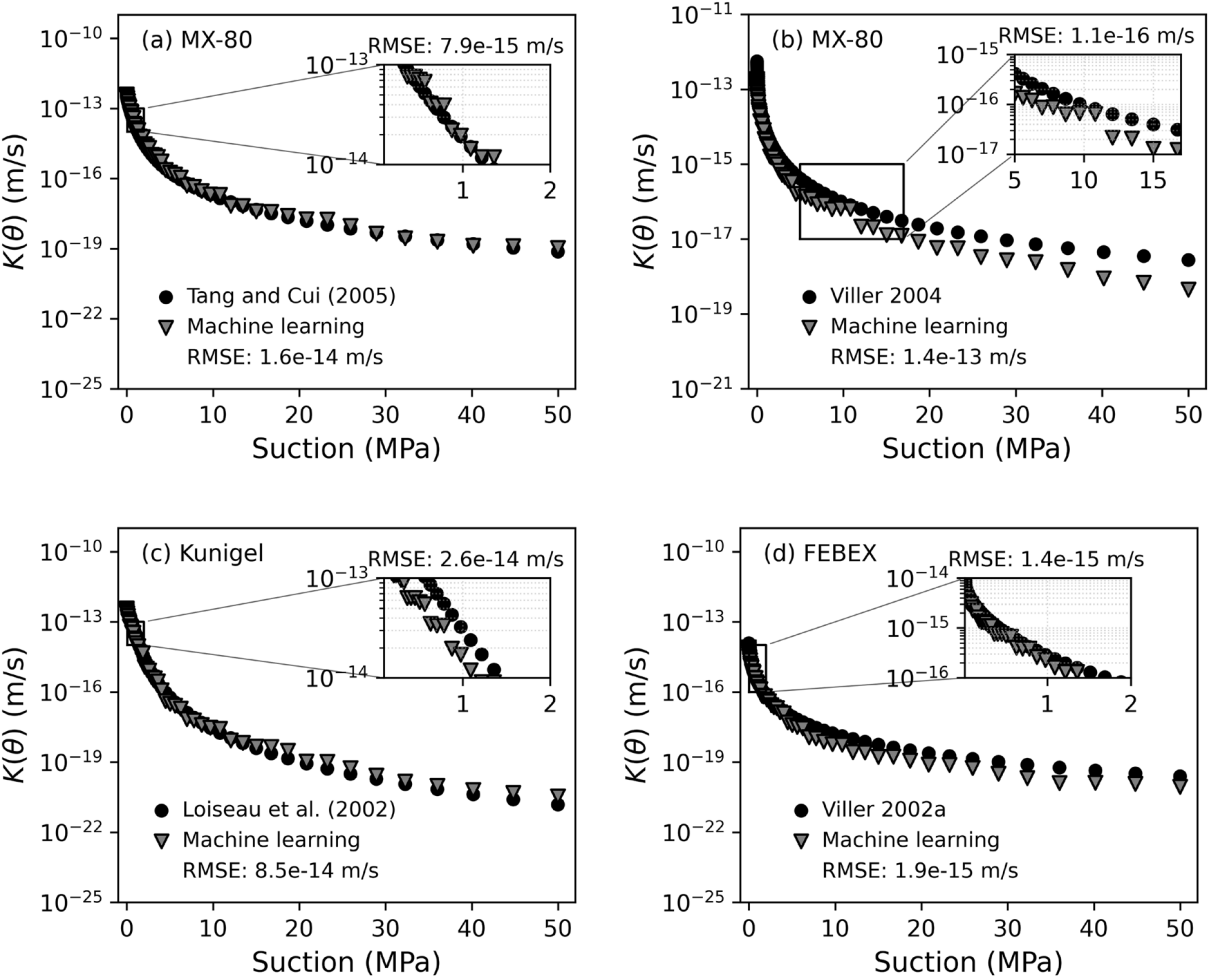
Performance analysis of machine learning model in predicting *K(θ )*: (**a**) MX-80 bentonite^29^; (**b**) MX-80 bentonite^30^; (**c**) Kunigel bentonite^34^; (**d**) FEBEX bentonite^27^.

The machine learning model developed to predict the unsaturated hydraulic conductivity *K(θ )* was trained on hybrid dataset that combines experimental measurements with physically consistent simulated targets. This physics-guided augmentation strategy enabled the model to learn the unsaturated hydraulic conductivity function across several orders of magnitude. To evaluate the performance of the developed model, its predictions are compared with those obtained from the Van Genuchten model (Figure 3). The machine learning model uses input features consisting of experimental soil properties, boundary conditions (confined/unconfined), hydraulic paths (wetting/drying), and simulated random suction values. The Van Genuchten model (Equation 1) was employed to generate the target *K(θ )* values. Although the reference *K(θ )* curves were generated using the Van Genuchten model, the corresponding model parameters were obtained by fitting experimentally measured SWRCs. The required $K_s$ value for each soil sample to anchor the unsaturated conductivity curve was obtained from the developed data-driven machine learning model.

Figure 3(a) shows the comparison between machine learning predictions and reference *K(θ )* for MX-80 bentonite under confined drying conditions. This MX-80 bentonite has a very high plasticity index (*PI = 484*), a void ratio of 0.64, and an initial dry density of 1.68 g/cm^3^, representing a densely compacted state. The developed model accurately captures the sharp decrease in hydraulic conductivity at low suction, reflecting the transition from capillary to adsorbed water flow in a confined microstructure. At higher suctions, the predicted trend remains consistent with the Van Genuchten showing a global RMSE of $1.6 \times 10^{-14}$ $\hbox {m s}^{-1}$.

Figure 3(b) presents the comparison for MX-80 bentonite under confined conditions along the wetting path. This bentonite has high plasticity index (*PI = 390*), a void ratio of 0.58, and an initial dry density of 1.60 g/cm^3^, corresponding to a dense compaction state. Slight deviations are observed at high suction levels, where the bentonite is effectively in a dried state and hydraulic conductivity is extremely low. As the machine learning model was predominantly trained using drying-path data, such small deviations are expected. A minor local discontinuity around 10 MPa is also observed; however, the overall trend and curve shape are well reproduced. The low global RMSE ($1.4 \times 10^{-13}$ $\hbox {m s}^{-1}$) shows the model’s capability to replicate wetting-induced hydraulic conductivity behaviour in compacted MX-80 bentonite.

Figure 3(c) presents the comparison for Kunigel-V1 bentonite under confined conditions along the wetting path. This bentonite mixture has a low void ratio of 0.34, and a high initial dry density of 2.03 g/cm^3^, indicating a very dense compaction state. The sample is a mixture of 70% bentonite and 30% sand, and has comparatively low montmorillonite content of 33.6%. Slight deviations appear at high suction levels, where hydraulic conductivity values approach the measurement limit. Nevertheless, the model reproduces both the overall trend and magnitude of the response well, showing its robustness and reliability in capturing the hydraulic behaviour of mixed bentonite materials.

Figure 3(d) presents the comparison for densely compacted FEBEX bentonite under confined conditions. This bentonite is characterized by high montmorillonite content. The machine learning model accurately captures the rapid decrease in hydraulic conductivity at low suction, associated with the progressive desaturation of the macro-pore network. At higher suction levels, where water flow is governed primarily by adsorbed films, the predicted curve remains smooth and consistent with the reference behaviour. Only minimal deviation is observed near the lowest conductivity range, where measurements approach the experimental resolution limit. The very low global RMSE of $1.9 \times 10^{-15}$ $\hbox {m s}^{-1}$ confirms the model’s strong predictive capability and its robustness in reproducing the unsaturated hydraulic response of highly compacted FEBEX bentonite.

## Hybrid model validation

The training targets of the proposed machine learning model were generated using the Van Genuchten model. The Van Genuchten formulation predicts a monotonic decrease in unsaturated hydraulic conductivity with increasing suction and is primarily applicable to continuous porous media in which flow is governed by capillary processes. However, several laboratory studies report a different response at high suction levels, characterized by a non-monotonic, U-shaped relationship between hydraulic conductivity and suction^49,50^. Specifically, *K(θ )* decreases with increasing suction up to a critical point and then increases again under very high suction. This apparent increase is mainly attributed to the formation of desiccation-induced microcracks and fractures within compacted bentonite specimens^51,52^. These structural discontinuities create preferential flow paths that dominate the hydraulic response, yielding conductivity values that no longer reflect intrinsic pore-scale transport within the intact clay matrix. Although this behaviour is physically meaningful for damaged or cracked samples, it corresponds to a transport mechanism that differs fundamentally from the continuum flow assumed in models such as the Van Genuchten model. Consequently, such data cannot be represented by the Van Genuchten model. In contrast^12^, developed a purely data-driven machine learning model trained directly on experimental unsaturated conductivity data, which successfully reproduces this U-shaped behaviour.

However, the physics-guided machine learning model developed in this study is trained to reproduce the monotonic decay of unsaturated hydraulic conductivity and is intended for bentonite considered in a crack-free state. Bentonite has a pronounced swelling capacity, and crack healing is expected due to the osmotic swelling of montmorillonite^51^. Under these conditions, flow is governed by capillary processes within a continuous pore network, and the Van Genuchten model remains physically appropriate.

To validate the proposed model, Korean Ca-type Gyeongju bentonite^53^ was selected. This bentonite type was excluded from both the training and testing datasets, ensuring a strictly blind validation scenario. Available soil index properties of this bentonite are summarized in Table 4. Several inputs required by the developed machine learning models were not reported in the original study and were therefore assigned representative laboratory values (e.g., $W_i = 10\%$, $T = 23^{\circ }$C), consistent with standard bentonite testing conditions. The initial void ratio was computed from $G_\text {s}$ and $\rho _\text {d}$. Although these assumptions introduce some uncertainty, they reflect realistic experimental practice^54^.

In practice, unsaturated hydraulic conductivity is commonly obtained through the following sequence: (i) experimentally determine water content at different suction levels and measure the saturated hydraulic conductivity, (ii) fit the Van Genuchten model to the SWRC data, and (iii) use the fitted parameters in Equations 2 and 1 to compute *K(θ )*.

^53^ conducted a laboratory experiment and determined volumetric water content over a wide suction range. Following the traditional approach, the Van Genuchten model was fitted to the experimental data using the same procedure applied to the 46 SWRC datasets in this study. The fitting performance was excellent, with an RMSE of 0.0049 and an $R^2$ of 0.997. The saturated hydraulic conductivity $K_s$ was not reported and was therefore predicted using the developed data-driven model. The predicted value ($K_s = 6.71 \times 10^{-14}$ $\hbox {m s}^{-1}$) lies close to the mean of the compiled bentonite dataset (Table 1). The fitted Van Genuchten parameters and the predicted $K_s$ were then used in Equations 2 and 1to compute the reference conductivity curve^55^.

The developed physics-guided machine learning framework emulates this traditional approach while removing its labor-intensive steps. The model predicts *K(θ )* directly from readily available soil properties (see Table 4), while remaining consistent with the same constitutive structure. Notably, the model does not require $K_s$ as an explicit input.

**Fig. 4:**
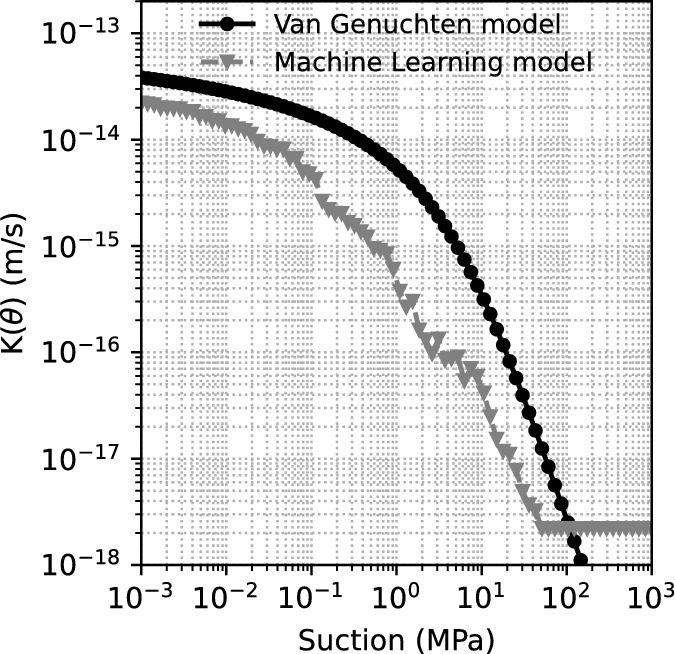
Comparison between the fitted Van Genuchten model and the developed machine learning prediction of the unsaturated hydraulic conductivity of Korean Ca-type Gyeongju bentonite^53^.

Figure 4 compares the traditionally computed *K(θ )* using Van Genuchten model with the prediction of the developed machine learning model. Although this bentonite was not part of the training set and several inputs were assumed, the machine learning model reproduces a *K(θ )* curve that is consistent with the Van Genuchten response. Both the magnitude and the overall curvature of the conductivity–suction relationship are captured over several orders of magnitude. Differences at high suction arise from uncertainty in the reconstructed inputs and from the strong sensitivity of desaturation behaviour to the initial state variables. The generally close agreement indicates that the developed model generalizes well to unseen bentonite and yields physically coherent predictions.

**Table 4:** Key soil properties, experimental settings and boundary conditions of the Korean Ca-type Gyeongju bentonite used as inputs for the developed models to predict $K_s$ and *K(θ )*.

**Input variable**	$K_s$ ** model**	*K(θ )* ** model**
Montmorillonite content, $\text {Mt.c}$ (%)	61.7	61.7
Specific gravity, $G_\text {s}$ (–)	2.71	2.71
Liquid limit, $w_\text {L}$ (%)	146.7	–
Plastic limit, $w_\text {P}$ (%)	28.4	–
Plasticity index, ($I_\text {p}$ (%)	–	118.3
Initial water content, $w_\text {i}$ (%)	10 (assumed)	10 (assumed)
Dry density, $\rho _\text {d}$ (g $\hbox {cm}^{-3}$)	1.73	1.73
Temperature, $T (^\circ \hbox {C}$)	23 (assumed)	–
Initial void ratio, $e_0$ (–)	–	0.57 (calculated)
Boundary condition	–	Confined (1)
Hydraulic path	–	Drying (0)
Suction, *ψ* (MPa)	–	$10^{-3}$–$10^{3}$
Output	$K_s = 6.71 \times 10^{-14}~\hbox {m s}^{-1}$	see Figure. 4

## Limitations

The developed machine learning models effectively predicts saturated and unsaturated hydraulic conductivity of bentonite within the range of soil conditions represented in the compiled dataset. However, the unsaturated conductivity model is trained primarily on synthetic targets generated from the Van Genuchten formulation, and its predictions are therefore constrained by the assumptions of that constitutive framework. In particular, the model assumes a monotonic decrease of hydraulic conductivity with increasing suction and does not represent crack-induced or non-monotonic flow behaviour that may occur in desiccated or structurally damaged specimens. The adopted formulation implicitly reflects a unimodal pore representation, whereas compacted bentonite is known to exhibit bimodal or multimodal pore structures that evolve with hydro-mechanical loading. Microstructural evolution, which strongly governs hydraulic response during wetting and drying, is not explicitly represented because the available inputs describe macroscopic soil properties rather than time-dependent pore-scale processes.

Beyond the limitations associated with the available dataset, the representation of hydraulic loading conditions^56^ in the proposed model is also simplified. Although binary indicators are used to distinguish wetting/drying paths and confined/unconfined boundary conditions, they do not explicitly represent the complete hydraulic history, scanning hysteresis loops, stress evolution, or deformation.

In addition, although a stratified 80:20 train-test split was adopted to preserve the proportion of confined and unconfined samples, the predictive performance under unconfined conditions is inherently constrained by the limited diversity of the underlying experimental data. In particular, the unconfined drying path is represented by only one experimentally measured SWRC (Table 2), and therefore predictions for unconfined conditions should be interpreted with appropriate caution. Future work could focus on time-resolved experimental monitoring of bentonite microstructure together with simultaneous measurements of unsaturated hydraulic conductivity. Such datasets would enable the development of machine learning models that explicitly learn the evolution of hydraulic behaviour driven by microstructural changes.

## Summary and conclusions

This study developed a data-driven machine learning model to predict saturated hydraulic conductivity and physics-guided machine learning models to predict unsaturated hydraulic conductivity of bentonite. To predict unsaturated hydraulic conductivity, this study integrates experimentally measured data, synthetic data generated from fitted Van Genuchten models, and the predicted saturated hydraulic conductivity from the developed data-driven model. This novel approach enhances predictive capability across a wide range of soil hydraulic conditions. The main findings and conclusions of this study are summarized as follows:

- A data-driven machine learning model was developed to predict the saturated hydraulic conductivity ($K_s$) of bentonite using key soil index parameters. The Catboost training algorithm achieved excellent predictive accuracy, with a test-set performance of $R^2 = 0.992$ and $\textrm{RMSE} = 0.503$ in $\hbox {log}_{10}(K)$ space, showing strong generalization across different bentonite types and compaction states.
- A machine learning model was developed to predict the unsaturated hydraulic conductivity *K(θ )*. The Catboost training algorithm shows outstanding performance, reaching a test-set accuracy of $R^2 = 0.994$ and $\textrm{RMSE} = 0.202$ in $\hbox {log}_{10}(K)$ space, enabling reliable prediction of hydraulic conductivity over several orders of magnitude and across the full suction range.
- A novel hybrid framework was introduced by coupling the predicted $K_s$ from the data-driven model with synthetic hydraulic data generated from the fitted Van Genuchten formulation. This integration embeds physical consistency into the learning process and allows the unsaturated model to inherit both experimental realism and theoretical continuity. As a result, the model accurately reproduces the sharp conductivity drop near air-entry and the gradual decay at higher suctions. This strategy significantly enhances robustness and ensures physically meaningful predictions across more than fourteen orders of magnitude in hydraulic conductivity.
- A strictly blind hybrid validation was performed using Korean Ca-type Gyeongju bentonite. The physics-guided machine learning model reproduced the Van Genuchten-consistent conductivity curve using only soil index inputs, despite the partial assumption of missing input parameters. The close agreement with the traditionally fitted hydraulic response confirms that the model generalizes to unseen bentonite and preserves physically coherent behaviour while eliminating the need for labor-intensive experimental procedures.

The deployment of the developed models through accessible web-based platforms further enhances their practical applicability, enabling rapid estimation of hydraulic conductivity without the need for laboratory testing or specialized computational skills. This facilitates their direct use in engineering practice and supports efficient decision-making in the design and assessment of barrier systems. The applicability of the unsaturated hydraulic conductivity *K(θ )* model under unconfined conditions remains limited by the small number of experimentally measured SWRCs, particularly for the drying path. Consequently, predictions under unconfined conditions should be restricted to the training domain and interpreted with caution.

### Model availability

The developed data-driven and physics-guided machine learning models are publicly available through dedicated web-based user interfaces, allowing researchers and practitioners to directly predict the saturated and unsaturated hydraulic conductivity of bentonite using the required soil parameters.

The interface for the saturated hydraulic conductivity model is available at: https://huggingface.co/spaces/Muntasir006/saturated-hydraulic-conductivity-of-bentonite-clay

The interface for the unsaturated hydraulic conductivity model is available at: https://huggingface.co/spaces/Muntasir006/Unsaturated_hydraulic_conductivity_of_bentonite

The developed models are intended for monotonic hydraulic conductivity behaviour represented by the training data and are not applicable to cracking-induced or other non-monotonic flow behaviour in bentonite. The web-based implementations are intended to support rather than replace engineering judgment. Users should ensure that predictions are made within the validated applicability domain of the proposed models, and estimates outside this domain or under data-scarce conditions, such as unconfined drying, should be interpreted with appropriate caution.

## Data Availability

The datasets generated and/or analyzed during the current study are available from the corresponding author upon reasonable request.
